# Video-thoracoscopic enucleation of esophageal leiomyoma

**DOI:** 10.1186/1477-7819-10-52

**Published:** 2012-03-16

**Authors:** Shi-Ping Luh, Sheng-Mou Hou, Chien-Chung Fang, Chi-Yi Chen

**Affiliations:** 1 Departments of Surgery, Shin Kong Wu Ho-Su Memorial Hospital, 95 Wen Chang Rd, Shih Lin, Taipei City (111), Taiwan; 2 Department of Gastroenterology, Chiayi Christian Hospital, 539 Chung-Shiao Rd, Chiayi City (600), Taiwan

**Keywords:** esophageal leiomyoma, video-assisted thoracoscopic surgery, enucleation

## Abstract

**Background:**

Leiomyoma is the most common benign tumor of the esophagus. Surgical enucleation is indicated in case of symptoms or an unclear diagnosis, and open thoracotomy has long been the standard approach for this procedure. However, enucleation through video assisted thoracoscopic surgery (VATS) has been developed as a preferred approach for most lesions in recent years.

**Method:**

Herein we report our twelve patients (seven men and five women, with median age of 42 years) from 2001 to 2009, who underwent enucleation through VATS for esophageal leiomyomas, with a size from 1 to 8 cm in diameter (median: 5), and at different locations, from the thoracic outlet to near the diaphragmatic level of the thoracic esophagus. Intraoperative fiberoptic esophagoscopy was performed in two patients for localization by illumination. A right-sided approach was performed in eight cases (upper two thirds of esophagus) and the left-sided in another four cases (lower third of esophagus).

**Result:**

The median operative time was 95 minutes (70 to 230 minutes). Four of them required small utility incisions (4-6 cm) for better exploration and manipulation. There were no major complications, such as death or empyema due to leaks from mucosal tears, and the presenting symptoms were improved during the follow-up period, from 12 to 98 months.

**Conclusion:**

VATS can be considered as an initial approach for most patients with esophageal leiomyomas, even large in size, irregular in shape, or at unfavorable location. It is a safe, minimally invasive, and effective treatment. However, conversion to open thoracotomy should be required for the sake of clinical or technical concern.

## Background

Benign tumors of the esophagus are uncommon, and the incidence varies from 0.005% to 5.1% in autopsy cases; and from less than 1% to 10% in all esophageal neoplasms [1-3]. Leiomyomas constitute 70-80% of all benign esophageal neoplasms [1,3-5]. Other benign esophageal tumors such as gastrointestinal stromal tumors, granular cell tumors or schwannomas are extremely rare. Esophageal leiomyomas are usually found in patients between 20 and 50 years of age, with an about twofold more male predominance commonly occurring in the lower two-thirds of esophagus [4,5]. Most of these patients are asymptomatic. Dysphagia is the most common symptom, followed by chest tightness and pain. The treatment strategy for esophageal leiomyomas is to continue follow-up dealing with smaller tumors and to do a surgical resection of larger or symptomatic tumors. The traditional open thoracotomy for enucleation of this tumor has been gradually replaced by minimally invasive thoracoscopic or laparoscopic approaches since 1992 [1,3,6-12]. Video assisted thoracoscopic surgery (VATS) is selected for patients with leiomyomas located in the upper two-thirds of thoracic esophagus. However, either VATS or laparoscopic approaches have been reported to be used in patients with leiomyomas in the lower thirds of the esophagus [9,10]. Herein we present our experiences with patients having esophageal leiomyomas, who underwent surgical enucleation successfully by VATS.

## Methods

**Figure 1:**
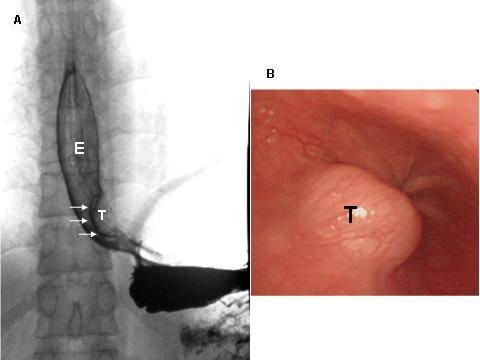
**A. Esophagogram of Case 10**. Reveals typical findings of intramural tumor (T) near the esophago-gastric junction: smooth surface, clear-cut margins, and sharp angles at upper and lower ends of the tumor. E: esophagus. B. Endoscopic view of the esophageal leiomyoma (T). (Case 10).

**Figure 2:**
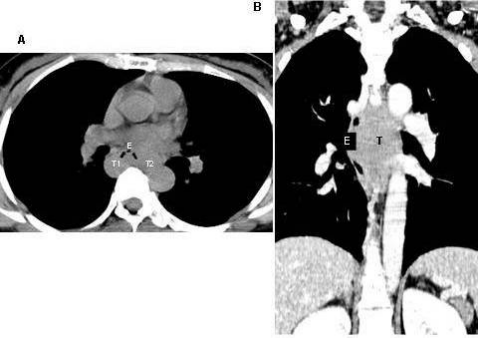
**Computed tomography, cross section (A)**. Reveals the esophageal lumen being compressed forwards by horse-shoe shaped (T1 and T2) leiomyoma. Coronal section (B). Reveals this tumor (T) compressing the esophagus (E) and adjacent structures of mediastinum. (Case 9).

From 2001 to 2009, a total of twelve patients with benign esophageal tumors were operated on through video-assisted thoracoscopic surgery (VATS) at our hospital. There were seven males and five females, with ages ranging from 26 to 65 years (median: 42 years). Surgical indications for enucleation were: symptoms of dysphagia, odynophagia, foreign body sensation on swallowing, and chest tightness; or the presence of a large sized tumor (≧ 4 cm). All of these above patients underwent detailed assessment, including a barium or urograffin swallowed esophagogram, an upper esophago-gastro-duodenal endoscopy, and computerized tomography (CT) of the chest (Figure 1 and 2). Six patients (50% of them) underwent endoscopic ultrasound (EUS) examinations. Endoscopic biopsies of the leiomyoma were taken on five patients (42% of them) because of the possibility that malignancy could not be excluded.

The minimal invasive surgical approaches for these patients included right VATS for tumors of the upper two-thirds of the esophagus, and left VATS for tumors of the lower third of the esophagus. Intraoperative endoscopy allowed for precise localization of the lesion for transillumination in two patients that had small lesions (1 cm and 1.5 cm in size). In one of our patients, Belsey Mark-IV, an anti-reflux procedure was added because the tumor was large (4 cm in size) and located near the gastro-intestinal junction.

**Figure 3:**
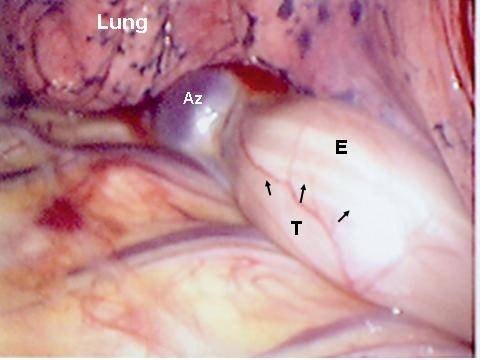
**Intraoperative view of the right thoracic cavity with an esophageal leiomyoma (T) close to the azygos vein (Az) and compressing the esophagus (E) anteriorly**. (Case 11).

**Figure 4:**
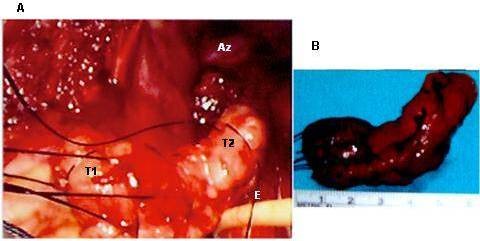
**A. Intraoperative view of the right thoracic cavity with a horse-shoe shape leiomyoma (T1 and T2) near the azygos vein (Az) removed from the esophagus (E)**. and B. surgical specimen of horse-shoe shape leiomyoma. (Case 11).

The VATS procedure is done under general anesthesia with double lumen intubation. The patient is placed in a right or left lateral decubitus position at about a 15° frontal incline. Three to four 10 to 12 mm cameras or working ports are placed over the chest wall, depending upon the location of the mass, and sometimes a working incision (without use of the spreader) is made about 3 to 4 cm in one of the ports, to facilitate the instruments manipulation. After the lesion is visualized by thoracoscopy or transilluminated by endoscopy, the mediastinal pleura over the tumor is incised longitudinally by an endoscopic hook electrocauterizer. Then the esophagus is circumferentially mobilized to facilitate exposure, and to avoid injuring the vagus nerve during this procedure. The mass is exposed after the overlying muscle is split longitudinally. The retracting suture is placed over the mass and then you must meticulously dissect the plane between the mass and the submucosal layer. The integrity of the mucosa must then be checked by observing if there is any bubble appearance in the water-submerged esophagus, after insufflating air through the nasogastric tube. The muscle layer is re-approximated and a 28 or 32 Fr chest tube is place through one of the ports (Figure 3 and 4).

## Results

The operative time ranged from 70 to 230 minutes (median, 95 minutes). All of these twelve patients had single lesion tumors and tumor size was in the range of 1 to 8 cm (median, 5 cm). The location, size and shape of the tumors are listed in Table 1. Eight of our patients with tumors in the upper two-thirds of esophagus underwent a right-sided VATS approach, and four of them with lower third esophageal tumors underwent a left sided approach. The operative course was uneventful for all of them. Postoperatively, a nasogastric tube was kept in place for three to seven days, until no evidence of leakage was found on the esophagrams that were performed on all of our patients postoperatively. A liquid diet for 24 hours was followed by a soft diet, given after the above assessment proved there was no leakage. The chest tube was removed in a range of between the 5^th ^to the 9^th ^postoperative day (median: 7 days). All twelve patients could have been discharged on the same day, but two of the twelve elected to stay in the hospital for one to two more days because of socio-cultural factors. No major complications, such as esophageal leakage, wound infection, or pneumonia, were found postoperatively in these patients. Histopathology confirmed benign esophageal leiomyoma with the appearance of uniform spindle cells, without abnormal mitotic figures. The immunohistochemical staining was positive for desmin and SMA, and negative for CD117 or CD34. All the patients were generally well and without dysphagia, gastro-esophageal reflux, tumor recurrence, or other related events during the twelve-months to eight-years of follow-up. At least 6 times of follow-up at the out-patient clinic for these patients were undertaken. Esophagogram, panendoscopy, or chest CT was used every three months postoperatively for these patients. Interview by telephone call was used for patients who were asymptomatic and were not willing to receive examinations in the clinic.

**Table 1:** Clinical data of 12 patients undergoing VATS enucleation of esophageal leiomyomas

Patient	Sex	Age years	Special procedure	Tumor location	Size (cm)	Shape	VATS approach	OpTime minutes	Post op Chest tube drainage duration (days)
1	F	43	Bx.	middle	4	Ov	Rt	200	7

2	M	32	Bx.	middle	5	Hs	Rt	85	5

3	M	47	Bx.	upper	3	Ov	Rt	100	7

4	F	41	Bx.	middle	4.5	Ov	Rt	150	5

5	M	26	EUS.	lower	2	Ro	Lt	85	5

6	F	50	Bx.	upper	3	Ov	Rt	90	5

7	M	30	EUS.	middle	4	Ov	Rt	120	6

8	M	53	EUS.	lower	2	Ro	Lt	85	6

9	M	65	EUS.	middle	8	Hs	Rt	230	9

10	F	27	Op-EGD	lower	1.5	Ro	Lt	75	6

11	F	34	Bx.	middle	7	Hs	Rt	210	7

12	M	49	EUS. Op-EGD	lower	1	Ro	Lt	70	6

Op-EGD: intra-operative esophagogastroduodenoscopy, EUS: endoscopic ultrasound.

Bx: endoscopic biopsy. Ov: ovoid, Ro: round, Hs: horse-shoe.

## Discussion

More than 90% of esophageal tumors are malignant. The leiomyoma is the most common benign esophageal tumor seen, and also very rarely seen. Other benign tumors of esophagus, such as lipoma or neurilemmoma, were extremely rare [12]. The reason for its occurrence remains unclear. Most leiomyomas of the esophagus arise from the muscularis propria, with some others from the muscularis mucosa [3]. Small tumors are usually asymptomatic, and are mostly round or oval-shaped. Larger tumors are more likely to be symptomatic, and will become lobular or horseshoe shaped, when surrounding the esophagus circumferentially in endoscopic or CT findings [4,13,14]. The most common location of the esophageal leiomyoma varied in different series, but was in the middle or lower third of the esophagus [1,4,13,14]. Esophagogastroscopy combined with endoscopic ultrasonographic evaluation of the tumor is mandatory in order to exclude cancer of esophagus from the differential diagnosis. Leiomyoma's typical appearance is of homogeneous and hypoechoic lesion with clear margin [9]. A contrast enhanced CT scan of the chest can demonstrate the extent of extraluminal involvement of the mass and exclude the possibility of malignancy. Endoscopic biopsy of submucosal or intramural tumors of the esophagus is not recommended because the subsequent mucosal defects or scarring will hamper surgical dissection and enucleation of the tumor [1]. However, sometimes this procedure is necessary to differentiate leiomyomas from other mesenchymal or epithelial malignancies such as leiomyosarcoma, gastrointestinal stromal tumors (GISTs) or carcinoma [15]. Five of our patients (42%) underwent this procedure pre-operatively. However, from our experience with previous studies a reliable diagnosis could not be obtained by this procedure. Our experience shows that the risk of mucosal perforation is higher if the biopsy was taken in deeper layers of the esophagus [1,3]. Differential diagnosis of benign or malignant esophageal mesenchymal tumors should be made by histopathological examination with immunohistochemical staining [16]. Both epitheloid and spindle cells can be seen in GISTs but only spindle cells can be seen in leiomyomas. There are about 20% of GISTs and 5% of leiomyomas diagnosed as malignant. Surgical therapy is usually indicated for patients with larger (≧ 5 cm) or symptomatic tumors [4]. However, we think that it can be useful in selected patients with smaller asymptomatic tumors because of its progressive growth in its natural course. There is also a possibility of malignancy and an indication for related minimally invasive surgery or endoscopic procedures [17].

The first successful resection and enucleation of a benign esophageal tumor was reported in 1932 and 1933 [18,19]. Surgical enucleations of esophageal leiomyomas by video assisted thoracoscopic surgery (VATS) have also been reported since 1992, and have contributed to a growing interest in use of this approach in recent years [6,20]. This minimally invasive approach has avoided morbidity associated with open thoracotomy, and such complications as wound pain and pulmonary atelectasis [21,22]. VATS is the preferred minimally invasive approach for enucleation of upper two-third leiomyomas of the esophagus, and laparoscopic approach can be an alternative to VATS for tumors of the lower third of esophagus [10,23]. Open surgical enucleation or even segmental esophagectomy is still required in some patients with giant esophageal leiomyomas [11,24]. There are some technical points to be considered during enucleation of the esophageal leiomyoma through VATS [1,21]. First, the operation should be postponed for at least two weeks after the patient undergoes endoscopic biopsy, to allow healing of the esophageal mucosa. Second, endoscopy can be used intra-operatively for localization of small tumors or for checking the mucosal integrity after enucleation. Third, doing a myotomy in the correct direction and right level, you must place traction sutures in the tumor so as to facilitate subsequent enucleation. An interrupted suture repair can be made through VATS if a small mucosa tear is noted. Fourth, no matter how large the esophageal leiomyoma is, enulceation should be done first. Esophagectomy should only be considered if the attempt at nucleation fails. Fifth, the approximation of the esophageal muscle after enucleation, should be performed to avoid possible motility disorders or pseudodiverticulum formation.

The choice of minimally invasive approaches for leiomyomas in the upper two- thirds of the esophagus are undoubtedly through VATS, but either laparoscopic [3,9,10] or VATS [1,23] approaches can be used for those tumors in the lower third of the esophagus. Though leiomyomas in the lower third of the esophago-gastric (EG) junction of the esophagus are not commonly accompanied by acid regurgitation or other motility disorders in some other series [3,9], only one of our patients was noted to have symptoms of heart burn. Procedures for motility or reflux disorders of the esophagus can also be performed through VATS [17]. Furthermore, less disturbance to the EG junction with lower prevalence of postoperative reflux disorders of the esophagus were found in patients undergoing VATS procedures during our experiences and prior reported series [1,23]. Thus we used the VATS approach for all leiomyomas located from the thoracic outlet to the EG junction of the esophagus.

Choosing to use the minimally invasive or open approach to treat esophageal leiomyomas depends on their size, shape and location. We have routinely tried the minimally invasive approach first, and would then convert to the open approach when there was a safety concern or technical limitation [7]. We have dealt with some cases of large or multiple leiomyomas of the esophagus prior to 2001 that required conversion to open thoracotomy. During the recent eight years we have successfully performed minimally invasive procedures through working ports or through small utility incisions (without spreader). The choice of enucleation or esophageal resection of esophageal leiomyomas also depends on their size, shape and location. There are no formal criteria for the selection of open or VATS approaches. From our experiences the esophageal leiomyoma of any size, shape or location can be tried to resect it through VATS at first. Conversion to open procedure should be considered in case of technical problems or limitations. We think that esophagectomy should only be considered when tumors fail to be enucleated [11,24]. Recently, the development of robot assisted esophagectomy through VATS, avoiding open thoracotomy, can be used for the treatment of giant leiomyomas of esophagus [25].

Our experiences in VATS treatment for esophageal leiomyomas have revealed satisfactory results with symptomatic relief, the least wound pain, and excellent wound cosmetics; and no major early or late complications have been found. VATS resections were feasible in our series, with some tumors challenging us to use minimally invasive techniques, when tumors were large in size, horseshaped, or were located in the azogoesophageal recess area. The median hospital length of stay in our series is longer than other series because the cost of the hospital stay in Taiwan is less expensive than in other countries, and we do not want to see complications resulting from too early oral feedings. We believe that all of our patients can take food orally about one to two days post-operatively.

## Conclusion

In conclusion, the resection of an esophageal leiomyoma at any location of the thoracic esophagus can be tried first through a VATS approach. Patients undergoing this minimally invasive procedure, according to our prior experiences and from literature, can benefit from limited operative trauma, less postoperative pain, more rapid recovery, shorter hospital stay and better wound cosmetics. Pre-operative endoscopic biopsy should be avoided unless a malignancy or mucosal lesion is suspected. Enucleation of a lower third esophageal leiomyoma through VATS procedure can result in less disturbance to the EG junction and related complications. Esophagectomy for a benign tumor of esophagus is not recommended unless enucleation fails to remove the tumor.

## Abbreviations

VATS: video assisted thoracoscopic surgery; CT: computerized tomography; EUS: endoscopic ultrasound; CD: cluster of differentiation; SMA: smooth muscle actin; GISTs: gastrointestinal stromal tumors; EG: esophago-gastric.

## Competing interests

All of the authors in this manuscript declared that no financial or non-financial interests were related to this study or future application after publication.

## Authors' contributions

SPL performed surgical management of all these cases and wrote the manuscript. (First Author). SMH supervised SPL to write this manuscript and gave comments to revise it (Corresponding Author). CCF performed upper GI scopy to find out some of these cases. CYC performed upper GI scopy to find out some of these cases. All authors read and approved the final manuscript.
